# Study on Experiment and Simulation of Shear Force on Membrane with Dynamic Cross-Flow for Lignin in Black Liquor

**DOI:** 10.3390/polym15020380

**Published:** 2023-01-11

**Authors:** Wenjie Zhao, Yu Wang, Qingdang Li

**Affiliations:** 1 College of Sino-German Science and Technology, Qingdao University of Science and Technology, Qingdao 266061, China; 2 College of Electromechanical Engineering, Qingdao University of Science and Technology, Qingdao 266061, China

**Keywords:** dynamic-cross-flow, filtration, lignin, membrane, simulation, shear force

## Abstract

To address the problem of lignin membrane fouling caused by dynamic cross-flow in the process of retaining and concentrating the black liquor byproduct of papermaking, this paper uses three different rotating structures (vane, disk and propeller) to increase the surface shear force and filtration flux of the membrane. In this paper, under different rotating speeds and different transmembrane pressure differences, numerical simulations were conducted on the shear forces generated by the three structures and the retention process on the surface of the membrane. The variation laws were also studied and compared. Under the same filtration conditions, the vane structure demonstrates better results than the propeller and disk structures in terms of increasing filtration flux. Based on the result, the vane shear force was simulated in terms of changing the particle deposition, and compared with vane rotating speeds of 100–700 r/min, the surface particle deposition of the membrane was significantly reduced at a rotating speed of 800 r/min. Finally, the numerical simulation results were experimentally validated to ensure the accuracy of the simulation. The findings provide a theoretical basis and practical value for solving the problem of lignin membrane fouling caused by dynamic cross-flow in the process of retaining and concentrating the black liquor byproduct of papermaking.

## 1. Introduction

The black liquor byproduct of papermaking contains a large number of pollutants such as lignin, sugar, hemicellulose, pigment and residual alkali. If discharged directly into the environment without treatment, it will lead to environmental pollution and damage to the natural ecology [1,2,3,4]. At the same time, lignin is an aromatic natural polymer with a three-dimensional (3D) space structure and multiple active radical groups formed of ether and carbon-carbon bonds, which give it excellent physical and chemical properties. When modified as a water treatment agent, it can not only help make full use of resources, and “turn waste into treasure”, but also realize the efficient treatment of wastewater [5,6,7,8,9,10].

There are many approaches to obtaining lignin, and lignin with different structures and properties can be recovered by means of different separation methods. Compared with traditional chemical separation technologies, such as acid, alkali and ionic liquid treatments, membrane technology has the advantages of low energy consumption and high energy conversion efficiency without a phase change in the separation process [11,12]. It can operate at room temperature and is suitable for the separation of heat-sensitive materials. In addition, with no need for additional materials to change the properties of the separated materials, it can save chemicals and conducts separation and concentration simultaneously, thus facilitating the graded recovery of valuable materials. Moreover, it boasts the advantages of simplicity, easy continuous operation and strong adaptability. Therefore, researchers tend to choose membrane separation for the separation of lignin from black liquor [13,14,15,16].

With the progress of the separation process, increased concentration of retention will lead to membrane fouling problems such as membrane blockage and concentration polarization, thus causing a decrease in the filtered flux [17,18,19,20]. In this regard, researchers found that increasing the shear force of the membrane surface is an effective method to decrease membrane fouling and increase filtered flux [21]. Wenjie Zhao et al. studied the treatment of paper-making black liquor by dynamic blade cross-flow membrane filtration equipment. A membrane with a molecular weight cut-off value of nanofiltration (NP010) demonstrates the best retention effect on black liquor, and the lignin content in the treated black liquor is 28% higher than that of untreated black liquor [22,23]. Roger Bouzerar et al. reported that the high shear force caused by disk rotation can prevent the formation of filter cake at low speed except for at the central part of the membrane [24]. C. Torras et al. studied the rotating structure of the disk, the relationship between the speed of the rotating disk and the average pressure on the membrane surface and the shear force on the membrane, respectively [25]. Kuo-Jen Hwang et al. used rotating disc dynamic microfiltration technology to concentrate microalgae and discussed the effects of the disk rotating speed, feeding rate of the suspension liquid and transmembrane pressure (TMP) on filtered flux and cake performance [26,27,28,29,30].

Based on the above research, this paper used numerical simulations to investigate the influence of vane, disk and propeller structures on shear force on the membrane surface, particle deposition and flux variation under different transmembrane pressure differences and rotating speeds, and different inlet and outlet positions of the pulping black liquor. The simulation results have been validated by experiments.

## 2. Numerical Simulation Methods

### 2.1. Research on Simulation Theory

As the heat exchange and temperature field in the whole process were negligible, the energy conservation equation was not considered in the simulation. Instead, the mass conservation equation and momentum conservation equation were used. The RNG k-model under the turbulence model was selected for the numerical simulation of a dynamic cross-flow filter, which originates from the transient n-s equation and adopts an RNG mathematical method. The turbulence governing equation is defined as follows:

(1) $$\frac{\partial (\rho \varepsilon )}{\partial t}+\frac{\partial (\rho v_{j}\varepsilon )}{\partial x_{j}}=\frac{\partial }{\partial x_{j}}(\alpha_{\varepsilon }\mu_{eff}\frac{\partial \varepsilon }{\partial x_{j}})+C_{1\varepsilon }\frac{\varepsilon }{k}(G_{k}+G_{2\varepsilon }G_{b})-C_{1\varepsilon }\rho \frac{\varepsilon^{2}}{k}-R$$

In the equation, t is the time; ρ is the fluid density; k is the turbulent momentum; j is the direction coordinate component; $v_{j}$ is the direction velocity component; ε is the turbulent dissipation rate; $\alpha_{\varepsilon }$:ε is the reciprocal of the effective Prandtl number; $\mu_{\mathrm{eff}}$ is the equivalent viscosity; $G_{k}$ is the built items of turbulent momentum k due to average velocity gradient; $G_{b}$ is the built items of turbulent momentum k due to buoyancy; and R is an additional term, where $\eta =S\frac{k}{\varepsilon }$, $\eta_{0}$ = 4.83, β = 0.012; $C_{\mu }$ = 0.0845, $C_{1\varepsilon }$ = 1.42, $C_{2\varepsilon }$ = 1.68, $\alpha_{k}$ = 1.0, and $\alpha_{\varepsilon }$ = 0.769.

### 2.2. Geometric Model

**Figure 1:**
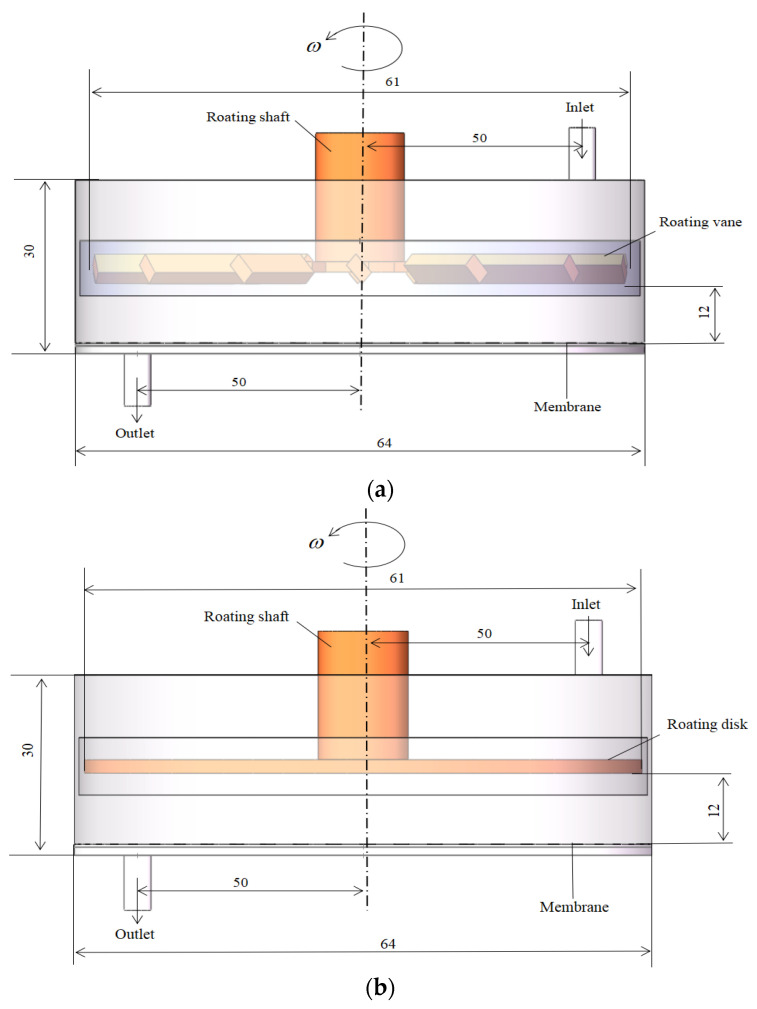

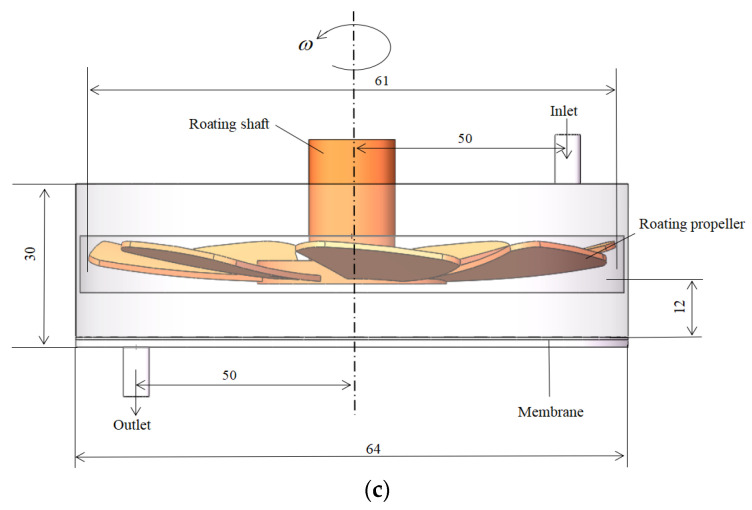
Numerical simulation model of three types of dynamic cross-flow filtration equipment.

Figure 1a–c introduces the plan of the three kinds of dynamic cross-flow filters made of 304 stainless steel: vane, disc and propeller. The filter diameter is 64 mm. Vanes, disks and propellers with a diameter of 61 mm are placed below the rotating shaft. The inlet and outlet are connected to the chamber for black liquor feed inflow and filtrate outflow. A circular membrane is installed at the bottom of the filter as a filter medium. The filtration area is $3.2\times 10^{-3}\;m^{2}$. The membrane is provided by BOKELA, a German company. The relevant data are shown in Table 1.

### 2.3. Numerical Model

**Figure 2:**
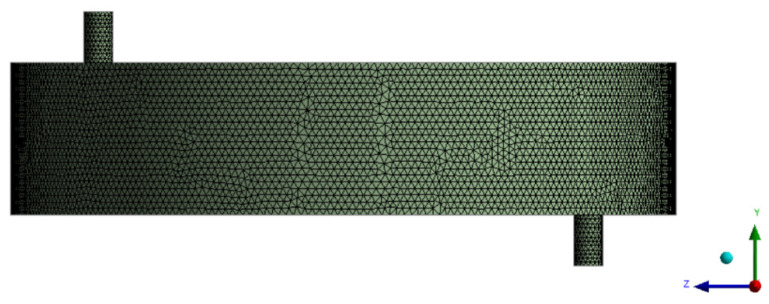
Overall mesh division diagram.

CFD software Fluen t(ANSYS Inc., version 16.0, Canonsburg, PA, USA) was used to simulate the flow field inside the filter chamber. The meshing module in ANSYS is adopted to create the mesh during the processing prior to simulation. As shown in Figure 2, the overall grid number is about 1,420,000, and the inspected quality is good.

**Figure 3:**
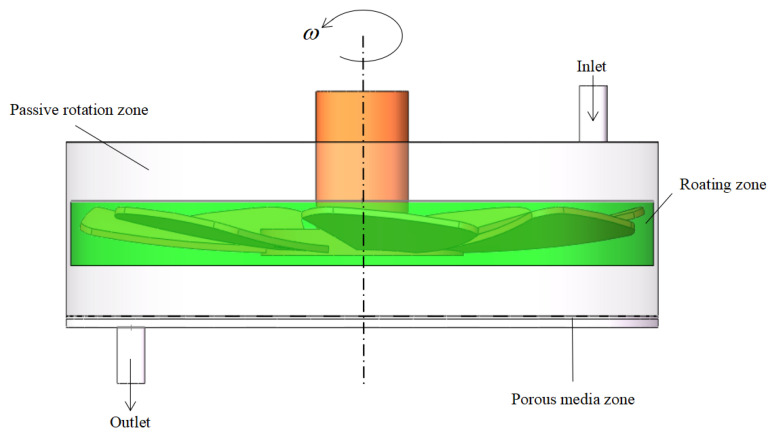
Settings of the geometric boundary type.

As shown in Figure 3, the fluid chamber is divided into three parts, the moving area surrounding the rotating structure, the porous medium area where the filter membrane is located, and other areas set as static areas. Local mesh encryption is conducted in the rotating dynamic region, which displays the apparent characteristic quantities and serves as a reference. The mesh independence verification is carried out for the characteristic quantities in the dynamic region, such as the velocity gradient, and the numerical difference is within 3%, which is an acceptable level. The SIMPLE algorithm is applied to couple the motion control equations. The boundary conditions include an inlet with a uniform velocity and a pressure outlet, which restrains the outlet backflow. The convergence criterion is that the continuous residual and velocity residual are both less than $1\times 10^{-6}$.

### 2.4. Setting of Geometric Boundary Type

The motion mode of the moving area is rotation, with the rotor speed increasing from 100 to 1000 r/min.

There is a pair of interfaces between the static area and the moving area, which are used for the transmission and transportation of physical quantities such as energy.

The porous medium model is set by Fluent software to simplify the membrane structure. According to the NP010 filter membrane simulated by the authors, the corresponding viscosity loss term and inertia loss term are set.

## 3. Simulation Results and Analysis

### 3.1. Shear Force on the Membrane Surface

**Figure 4:**
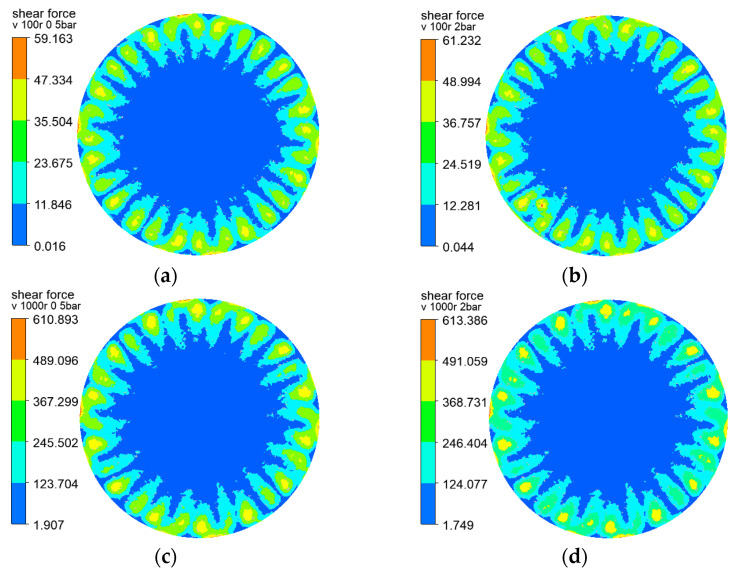
Cloud charts of shear force on the membrane surface when the vane is at different rotating speeds and pressures: (**a**) r = 100 r/min, △P = 0.5 bar (**b**) r = 100 r/min, △P = 2 bar (**c**) r = 1000 r/min, △P = 0.5 bar (**d**) r = 1000 r/min, △P = 2 bar.

**Figure 5:**
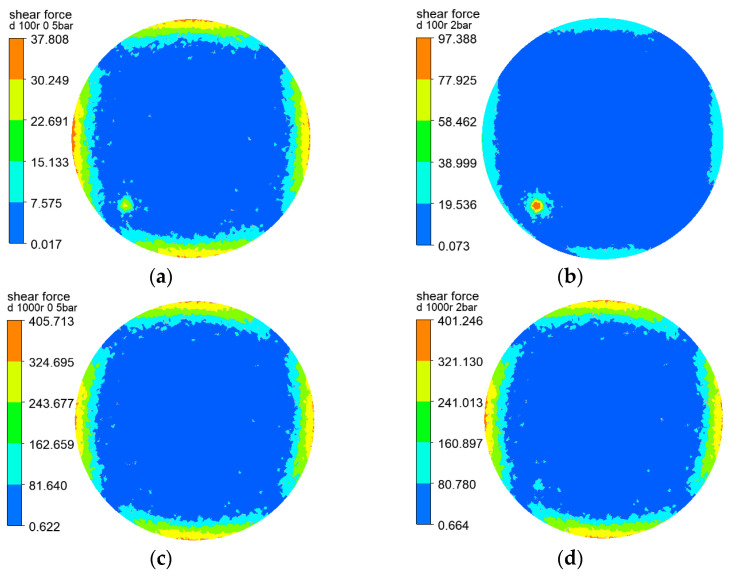
Cloud charts of shear force on the membrane surface when the disk is at different rotating speeds and pressures: (**a**) r = 100 r/min, △P = 0.5 bar (**b**) r = 100 r/min, △P = 2 bar (**c**) r = 1000 r/min, △P = 0.5 bar (**d**) r = 1000 r/min, △P = 2 bar.

**Figure 6:**
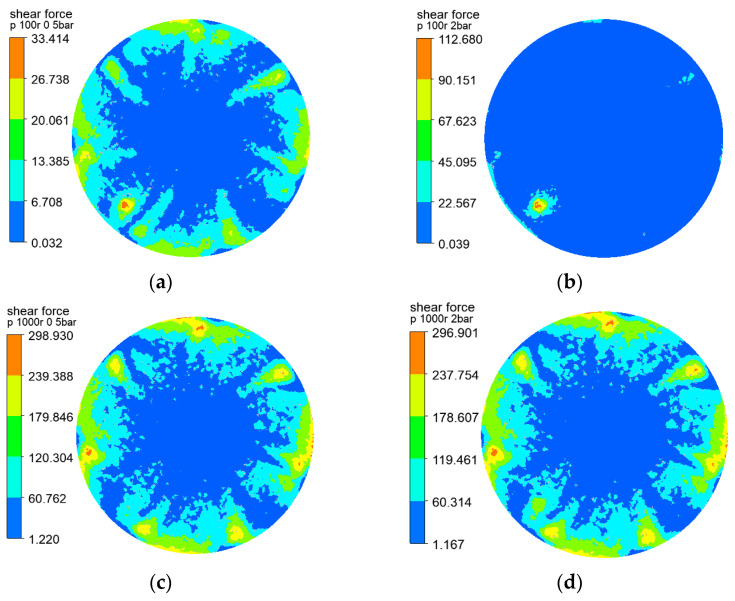
Cloud charts of shear force on the membrane surface when the propeller is at different rotating speeds and pressures: (**a**) r = 100 r/min, △P = 0.5 bar (**b**) r = 100 r/min, △P = 2 bar (**c**) r = 1000 r/min, △P = 0.5 bar (**d**) r = 1000 r/min, △P = 2 bar.

The shear forces of the three rotating structures on the membrane surface are shown in Figure 4, Figure 5 and Figure 6, and the results show that the distribution of the shear force on the membrane surface was largely consistent with the shape of the rotating structure. The shear force was observed to reach the lowest level at the center of the rotating structure, which facilitated the deposition and the formation of filter cake. The greater the velocity gradient, the greater the shear force caused by the outward rotating structure. In addition, the gap between adjacent rotating plates caused black liquor reflows—that is, the black liquor flowed downward in the vertical direction. At a rotating speed of 100 r/min, the transmembrane pressure difference varied slightly from 0.5 to 2 bar, and at a rotating speed of 1000 r/min, there was little change, which showed the influence of the transmembrane pressure difference on the shear force on the membrane surface. The sudden change in the lower left corner of the cloud chart was above the black liquor outlet, where the black liquor flowed out of the filtration equipment, and the large velocity gradient produced greater shear force than the surrounding flow field. The higher the rotating speed, the greater the shear force on the membrane surface, and the less likely the deposition and formation of filter cake. It can be seen that the rotating speed has a great influence on the shear force on the membrane surface.

### 3.2. Influence of Transmembrane Pressure Difference and Rotating Speed on Shear Force on the Membrane Surface

**Figure 7:**
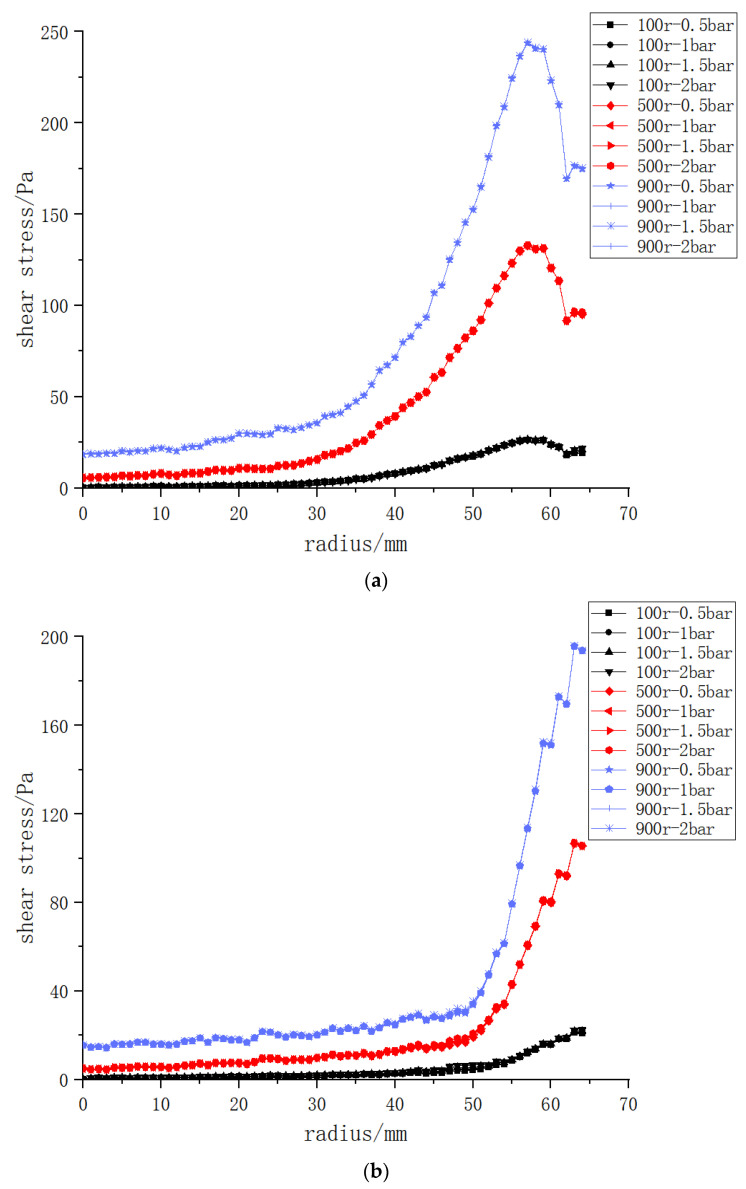

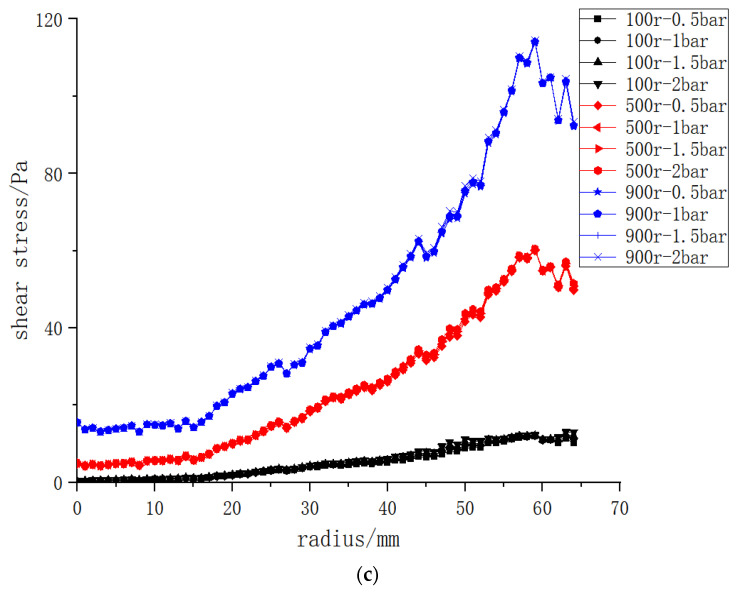
Cloud charts of shear force on the membrane surface of the three structures at different rotating speeds and transmembrane pressure differences: (**a**) vane (**b**) disk (**c**) propeller.

According to the theoretical study of dynamic cross-flow filtered flux, the transmembrane pressure difference and the rotating speed of the rotating structure are the influencing factors of the membrane filtered flux, while the membrane flux is related to the shear force on the membrane surface. As such, in order to further explore the influence of the transmembrane pressure difference and rotating speed on shear force on the membrane surface, the three rotating structures are set at rotating speeds of 100, 500 and 900 r/min, respectively, and the transmembrane pressure difference is set as 0.5 bar, 1 bar, 1.5 bar and 2 bar, respectively, to explore the shear force on the membrane surface. As shown in Figure 7, at the same rotating speed, the curves of shear force on the membrane surface produced by different transmembrane pressures are almost the same, and the influence of the transmembrane pressure difference was negligible. Rotating speed is the main factor affecting the shear force on the membrane surface.

### 3.3. Effect of Rotational Speed on the Shear Force on the Membrane Surface

After confirming that the transmembrane pressure difference has little effect on the shear force on the membrane surface, this paper mainly investigated the influence of the rotating speeds of the three structures on the shear force and compared the shear force at the same rotating speeds. The rotating speeds are 100, 200, 300, 400, 500, 600, 700, 800, 900 and 1000 r/min respectively, and the transmembrane pressure difference is kept constant at 0.5 bar.

**Figure 8:**
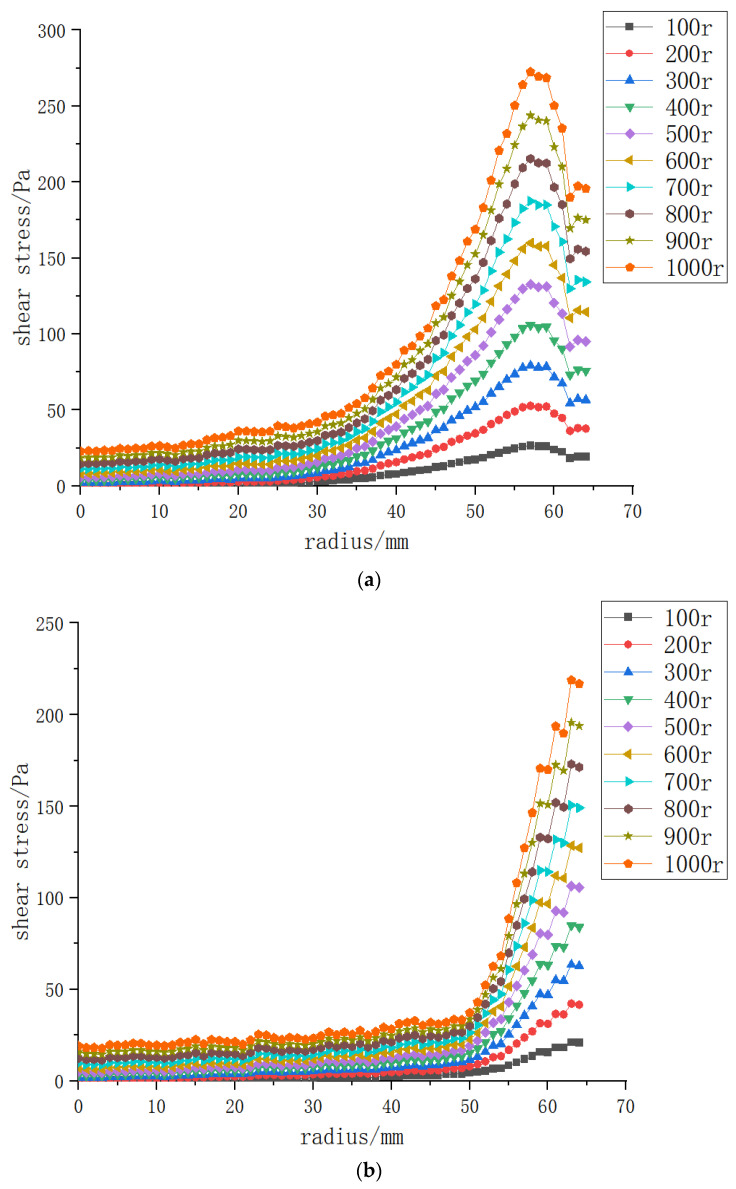

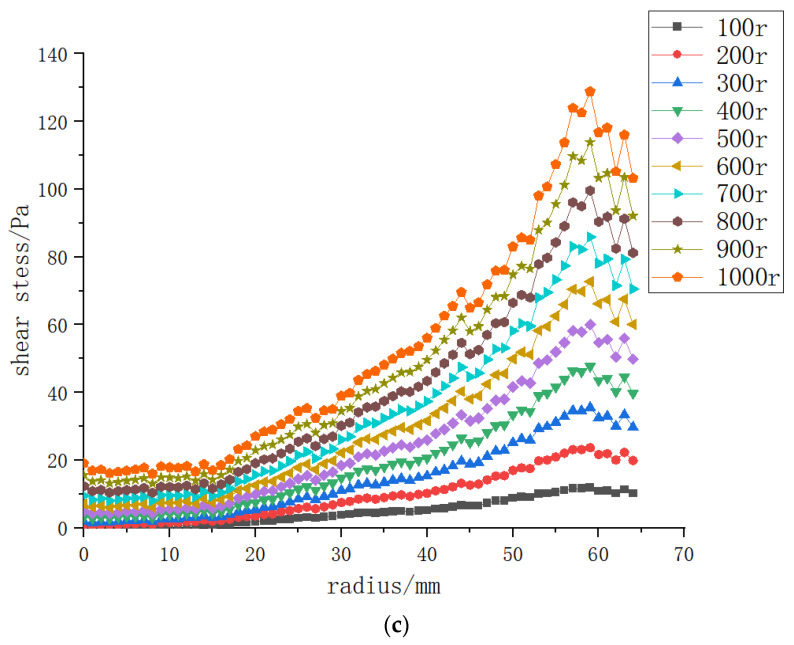
The shear force on the membrane surface of three structures at rotating speeds of 100–1000 r/min and a transmembrane pressure difference of 0.5 bar: (**a**) vane (**b**) disk (**c**) propeller.

As shown in Figure 8, the higher the rotating speed of the vanes, disks and propellers, the greater the shear force on the membrane surface; the shear force reached the lowest level at the center, and the bigger the radius, the greater the velocity gradient and the stronger the shear force. The shear forces of the three structures varied greatly from 45 to 55 mm, and the backflow velocity was relatively large at the black liquor inlet and outlet. Section 3.4 of this paper explores the influence of the location of the black liquor inlet and outlet on the shear force on the membrane surface. The shear force on the membrane surface began to fall continuously after 57 mm, which was about 89% of the radius. The shear force began to decrease from here to the wall due to the obstruction of the wall.

### 3.4. Effects of Different Rotating Structures on the Shear Force on the Membrane Surface

**Figure 9:**
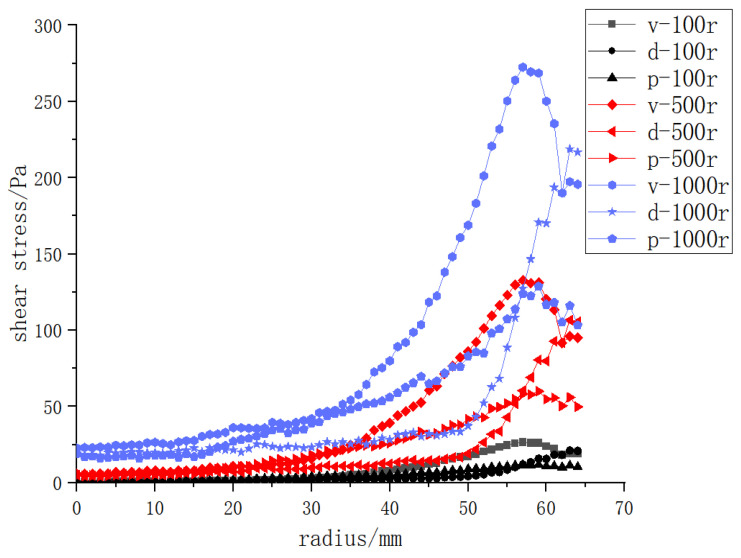
The shear force on the membrane surface at rotating speeds of 100, 500 and 1000 r/min; d: disk, p: propeller.

Figure 9 shows the comparison of the shear forces produced by the three structures at different rotating speeds on the membrane surface, and the shear forces produced by the vane at the same rotating speed are greater than those of the other two rotating structures. As the disc structure is continuous and centrosymmetric near the wall, it had a weaker backflow effect compared with the other two structures, thus generating greater shear force near the wall. However, under the same working conditions, the shear force produced by the vane structure was much stronger than that of the other two structures, delivering the best effect. This paper further explored the vane structure in the following sections.

Based on the fact that the vane rotating structure could bring better shear force on the membrane surface than the other two structures, this paper further investigated the influence of the position of the inlet and outlet distance from the central rotating shaft on the shear force on the membrane surface and carried out numerical simulation research with distances between the inlet and outlet of 50, 20 and 60 mm.

**Figure 10:**
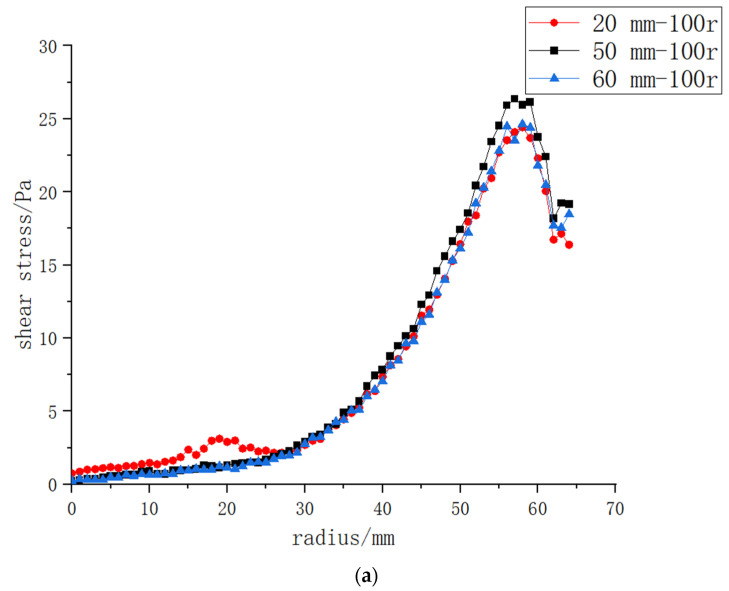

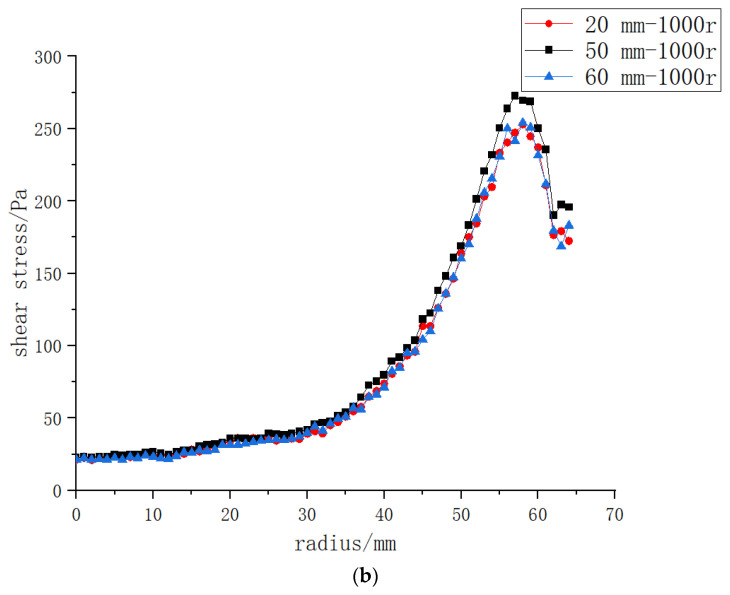
The shear force on the membrane surface when the vane structure is placed at different positions relative to the inlet and outlet, and at different rotating speeds: (**a**): 100 r/min; (**b**): 1000 r/min.

As indicated in Figure 10, at a low rotating speed of 100 r/min, a slight effect on the shear force on the membrane surface was observed 20 mm from the central axis at the inlet and outlet position, but the overall effect was negligible. The curves of 60 and 20 and 50 mm had little difference, and the shear force produced by a 50 mm separation was greater than that of 20 and 60 mm separations. At a rotating speed of 1000 r/min, the curves of the three distance positions showed little difference, and the value at 50 mm was higher than that at 20 and 60 mm. Generally speaking, the inlet and outlet positions had limited influence on the shear force on the membrane surface, and the value at 50 mm was higher than that at 60 and 20 mm.

### 3.5. Effects of Shear Force on Particle Deposition

After determining the influence of the optimal structure and preferable rotating speed on the shear force, this paper further investigated the influence of rotating speed and shear force on particle deposition. Black liquor contains most of the original cooking inorganic elements and the degraded, dissolved wood substance. The latter include acetic acid, formic acid, saccharinic acids, numerous other carboxylic acids (all as sodium salts), dissolved hemicelluloses (especially xylans), methanol and hundreds of other components. It is an extremely complex mixture. About seven tons of black liquor at 15% solids (about 10% organic chemicals and 5% inorganic chemicals with a total heat content of 13.5–14.5 MJ/kg solid is produced per ton of pulp) [31,32,33]. In other words, the initial solid content in paper-making black liquor is about 15%. In the numerical simulation, the speed was increased from 100 to 1000 r/min, and it was found that more particles were deposited at the outlet when the rotating speed ranged from 100 to 800 r/min because the shear force was not powerful enough to drive the particles away from the outlet. When the rotating speed was 800 r/min, fewer particles were deposited at the outlet, and a similar situation was observed at positions other than the rotating center. When the speed reached 800–1000 r/min, the same particle deposition situation was observed at the outlet, but the flux increased slowly and changed little with the continuous increase in rotating speed.

**Figure 11:**
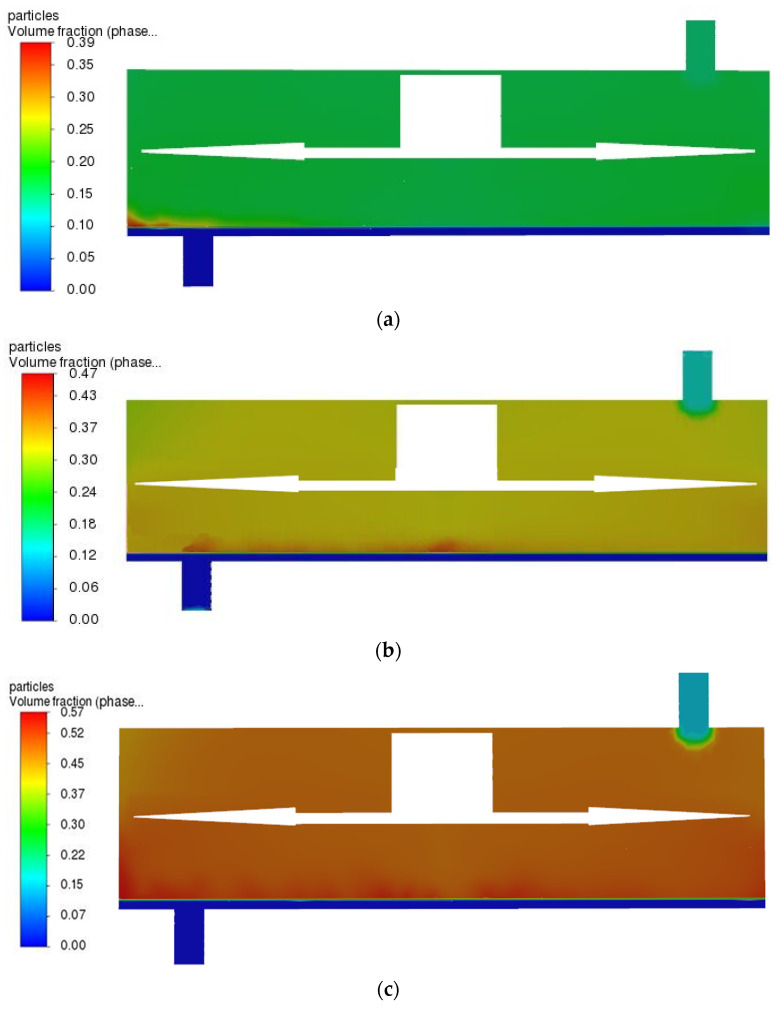
The particle phase distribution was simulated, showing the distribution stages of particles in the filter at different time t. (**a**) t = 2 s, (**b**) t = 20 s, (**c**) t = 36 s.

At a rotating speed of 800 r/min, the particle deposition on the membrane surface was significantly reduced compared with that at 100–700 r/min. Figure 11 shows the distribution of particles in the filtration equipment at the rotating speed of 800 r/min. Initially, 15% of the particles were uniformly distributed in the filter chamber, and when the filtration was in progress for 2 s, particles gathered near the outlet driven by the black liquor fluid. When the filtration continued until 20 s, the particles gathered at the place where the shear force on the membrane surface was the weakest generated by the rotating vane—that is, at the center of the rotating axis. Meanwhile, due to the retention of the membrane, the volume fraction of particles in the chamber further rose, and the retained lignin content increased when the filtration continued to 36 s.

## 4. Experimental Validation

From the data obtained in the experiment, the filtered flux was calculated according to the following Equation (2):

Filtration membrane flux:

(2) $$J=\frac{V_{p}}{t\cdot A_{M}}\left[\frac{l}{m^{2}\cdot h}\right]$$

J: membrane flux. Vp: filtrate volume. t: filtering time. A_M_: The area of the membrane. 

### 4.1. Laboratory Equipment and Materials

**Figure 12:**
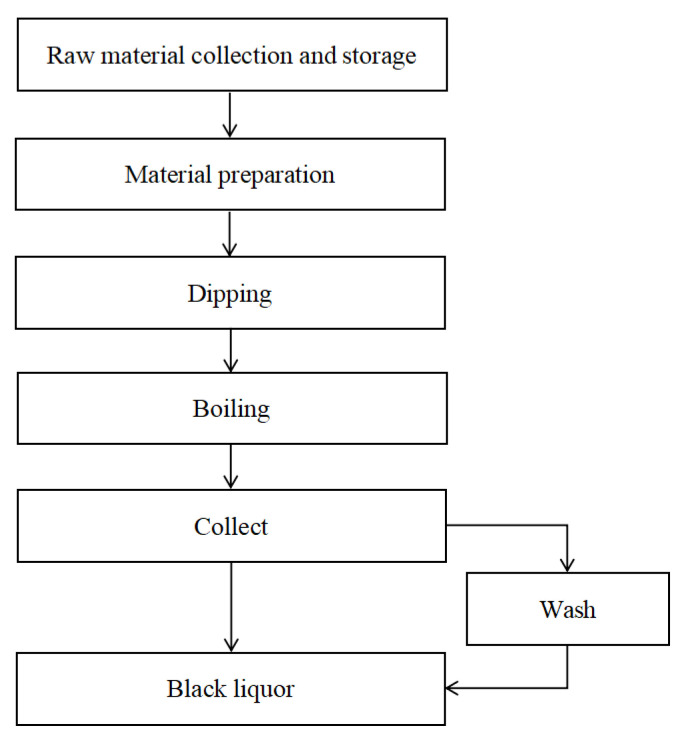
Flow chart of black liquid production.

The straw used in the experiment was purchased from Qingdao, China. After drying in air and storing for a period of time, the straw was cut into 3–5.5 cm sections and placed in a closed storage box for later use in dim light to balance the moisture. The experiment was conducted in a 3.5 L cooker in the laboratory. The black liquor preparation process is shown in Figure 12. In this study, ultrafiltration polyethersulfone polymer membrane NP010 was used. The alkaline pulping black liquor used in this study is shown in Table 2.

All the boiled pulp and black liquor were transferred from the cooker to the prepared storage box at room temperature. The black liquor and pulp were put into the centrifuge several times. After the original black liquor was centrifuged, the pulp was washed with distilled water three times before being centrifuged; then it was mixed with the original black liquor. Considering the size of the separation equipment, the extracted black liquor was divided into 41 parts on average, one of which was retained, and the lignin content of this part was measured as a reference. The remaining 40 black liquor samples were filtered using the membranes provided in Table 1. The transmembrane pressure was set at 0.5 bar, 1 bar and 1.5 bar, and the vane speed was 300 and 800 r/min.

### 4.2. Experimental Methods

**Figure 13:**
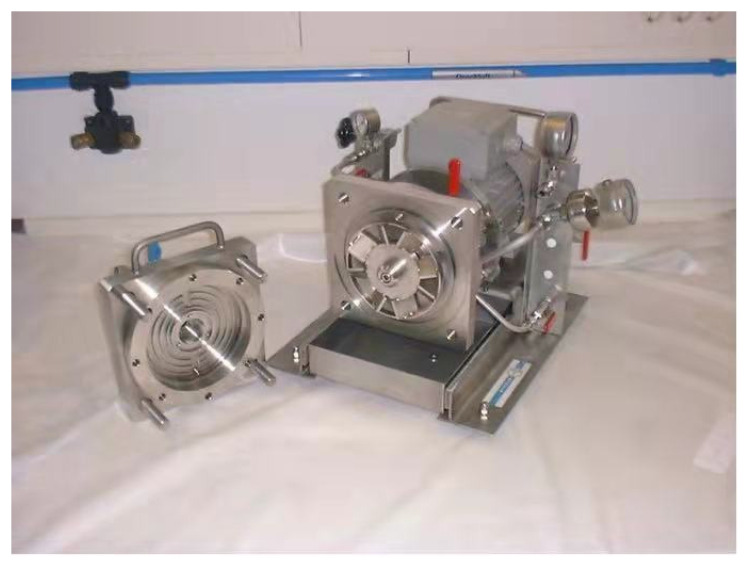
The dynamic cross-flow experimental setup, showing the vane.

**Figure 14:**
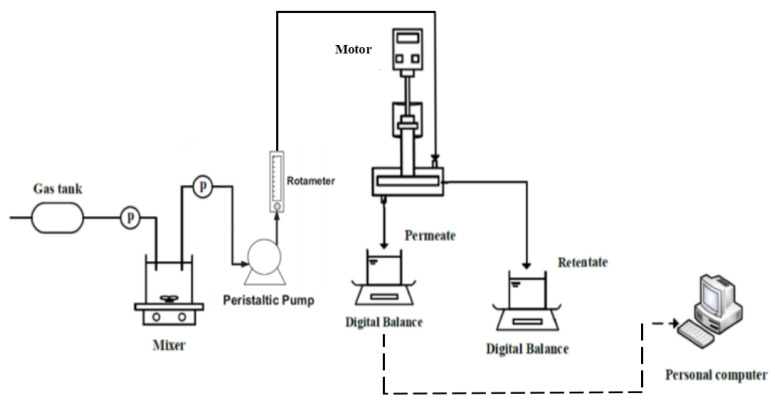
Flow chart of the dynamic cross-flow filtration process.

The dynamic blade cross-flow experimental equipment is shown in Figure 13, and the filtration flow chart is shown in Figure 14. As can be seen from Figure 14, the black liquor was stirred evenly by a propeller in a sealed barrel, then filtered and separated to avoid solute precipitation and compromising of the experimental effect. The black liquor containing lignin was retained, and the concentrate was recovered to the retention container. The filtrate was exudated under transmembrane pressure and collected in a penetrants container. The data in the filtration process were recorded by the computer.

### 4.3. Experimental Results

**Figure 15:**
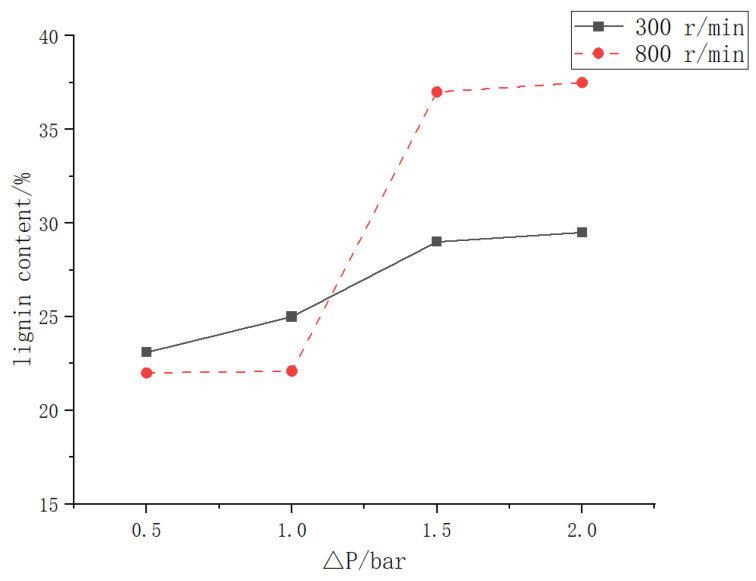
Changes in lignin content in black liquor filtered with vane stirring at 300 and 800 r/min.

The lignin content of black liquor prepared in the laboratory was measured to be 9%. After the vane-stirred dynamic cross-flow filtration experiment, the lignin content in the black liquor reached the levels shown in Figure 15. According to this curve, the lignin concentration in the filtered black liquor could be obtained.

**Figure 16:**
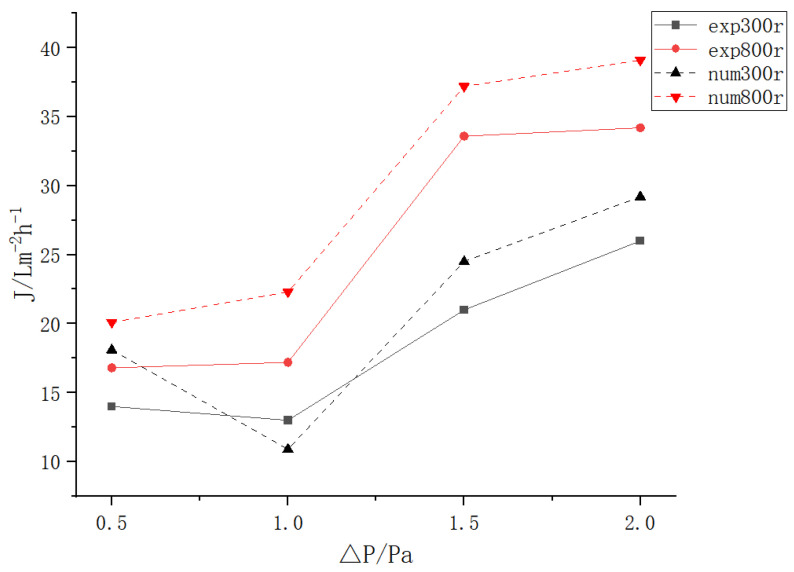
Diagram of the vane-stirred dynamic cross-flow experimental equipment flux.

In Figure 16, when the transmembrane pressure differences ranged from 0.5 to 1 bar, the flux showed no obvious change due to the insignificant transmembrane pressure difference. The flow direction of the dynamic cross-flow filtration liquid was perpendicular to the filtration direction, and the limited transmembrane pressure difference had little influence on the turbulent flow field inside the filter; when the speed was 300 r/min, the insufficient flow in the internal flow field led to inadequate energy transmission because of the low rotating speed, thus causing a local decrease in flux.

In addition, the numerical simulation results were largely consistent with the experimental data, demonstrating the applicability of the simulation results.

## 5. Conclusions

1. In dynamic cross-flow filter equipment, the rotating speed had the biggest influence on the shear force, and the higher the rotating speed, the stronger the shear force. With an increase in the radius, the shear force would increase, reaching a peak when the radius was 89% (57 mm), then the shear force would gradually decrease due to the backflow of the wall.

2. Under the same filtration conditions, the vane structure was superior to a propeller or disk in terms of increasing filtered flux. The disk structure could produce less shear force at the first 0–86% (55 mm) than the propeller, while it could produce more shear force at 87–100% than the propeller.

3. Under different rotating speeds and simulated transmembrane pressure differences, the effect of the three rotating structures on the shear force on the membrane surface was compared. The results showed that the vane structure could produce better shear force on the membrane surface than the disk and propeller structures. In particular, when the rotating speed was 800 r/min, particle deposition on the membrane surface was significantly reduced compared with that at 100–700 r/min.

## Figures and Tables

**Table 1:** Membrane information in the experiment.

Name	Membrane Material	Permeability [LMH/Bar]	PH Range	Nominal M.W.C.O (Da)
NP010	PES	≥5	0.0–14.0	1000–1200

**Table 2:** Cooking conditions for the black liquor.

Name	Cooking Temperature/°C	Cooking Time/Min	Effective Alkali/%	Solid-Liquid Ratio/*g/v*	PH
Soda-BL	110	90	15	1:10	12.7

## Data Availability

Data presented in this study are available on request from the corresponding author.
